# Metaplastic breast cancer: a clinicopathological analysis of nine cases highlighting diagnostic challenges and aggressive behavior

**DOI:** 10.3389/fonc.2026.1852513

**Published:** 2026-09-11

**Authors:** Yuan Shao, Binghua Kan, Yanni Li, Qing Zhou, Guiru Yan, Juhua Chen

**Affiliations:** 1 Department of Surgical Oncology, Hanzhong Central Hospital, Hanzhong, China; 2 Department of Endocrinology and Diabetes, Hanzhong Central Hospital, Hanzhong, China; 3 Department of Pathology, Hanzhong Central Hospital, Hanzhong, China

**Keywords:** breast cancer, metaplastic carcinoma, PIK3CA mutation, targeted therapy, triple-negative breast cancer

## Abstract

**Introduction:**

Metaplastic breast cancer (MBC) is a rare and highly heterogeneous subtype of breast cancer associated with limited treatment options and a poor prognosis. We aimed to characterize the clinical features, pathological findings, treatment patterns, and outcomes of patients with MBC.

**Methods:**

This single-center retrospective analysis included nine patients diagnosed with MBC at Hanzhong Central Hospital between January 2016 and July 2025. Clinical data, pathological features, treatment regimens, and follow-up outcomes were comprehensively reviewed.

**Results:**

Patient ages ranged from 26 to 76 years, with tumor diameters ranging from 1.5 cm to 7.0 cm, presenting as irregular hypoechoic masses on ultrasound. Immunohistochemical analysis revealed that the majority of cases (7/9, 77.8%) were triple-negative; one case (11.1%) was ER-low positive, and one case (11.1%) converted to HER2-positive following neoadjuvant therapy. All patients underwent mastectomy, with some undergoing additional lymph node dissection and radiotherapy. The most common metastatic sites were the liver and brain. At a median follow-up of 24 months (range: 15.7–28.4 months), five patients (55.6%) died from distant metastasis.

**Discussion:**

Preoperative diagnosis of MBC remains challenging and relies heavily on postoperative pathological examination. In this single-center series of nine patients, we observed aggressive clinical behavior characterized by a high rate of distant metastasis (liver and brain) and a short-term mortality of 55.6% (five of nine) at a median follow-up of 24 months. However, three patients remained disease-free, and one was alive with metastatic disease at the last follow-up, reflecting the heterogeneous clinical outcomes of this disease. The small sample size and the absence of molecular profiling preclude definitive conclusions regarding optimal treatment sequencing. Our findings underscore the diagnostic complexity, pathological heterogeneity, and urgent need for multicenter collaborative studies with integrated genomic characterization to guide future therapeutic strategies. The molecular data on PIK3CA mutation frequencies and targeted therapeutic approaches discussed in the context of the literature are derived from published studies and were not investigated in our cohort.

## 1 Introduction

Breast cancer remains the most commonly diagnosed malignancy among women globally, yet it ranks as the second leading cause of cancer-related mortality in this population, according to the 2022 global cancer data from the International Agency for Research on Cancer (IARC) (1). Among its diverse subtypes, metaplastic breast cancer (MBC) accounts for approximately 0.2% to 5% of all breast cancer cases (2). First described by Huvos et al. in 1973 (3), MBC was formally classified as a distinct solid tumor type by the World Health Organization (WHO) in 2000 (4), with the subsequent refinement of its histological classification based on stromal and epithelial components that was introduced in 2019 (5).

The histopathological spectrum of MBC includes several distinct subtypes, such as low-grade adenosquamous carcinoma, fibromatosis-like metaplastic carcinoma, squamous cell carcinoma, spindle cell carcinoma, heterologous mesenchymal differentiation (encompassing chondroid, rhabdoid, and osseous forms), and mixed-type metaplastic carcinoma (6). In addition to fibromatosis-like carcinomas and low-grade adenosquamous carcinomas, most metaplastic variants are characterized by aggressive clinical behavior, chemotherapy resistance, and elevated metastatic potential (7).

MBC typically affects middle-aged women, although some cases involve younger and older age groups. Compared with invasive ductal carcinoma (IDC), MBC is associated with older age at onset, larger tumor size, faster growth rate, and greater invasiveness (7–10). Patients with MBC are more likely to experience skin or chest wall invasion, although the incidence of axillary lymph node metastasis is relatively reduced (11, 12). Hematogenous dissemination is the predominant metastatic route, with some studies reporting the lungs and central nervous system as the most common sites of distant metastasis (13). Large population-based cohort analyses have further confirmed that metaplastic breast cancer is associated with worse long-term survival than conventional triple-negative breast cancer, even after adjustment for tumor stage (14). The prognosis of metastatic MBC remains poor, with a median overall survival time of 3–8 months (13).

In patients with MBC, the expression rates of estrogen receptor (ER), progesterone receptor (PR), and human epidermal growth factor receptor 2 (HER2) are relatively low, resulting in reduced efficacy of endocrine and targeted therapies (15). Recent genomic studies have significantly advanced our understanding of MBC biology. Genomic profiling of 123 MBC cases from a 2026 nationwide analysis identified TP53 (73.2%) and PIK3CA (46.0%) as the most frequent alterations (15). Notably, PIK3CA mutations have been associated with improved disease control in patients receiving taxane-based therapy (16). Complementary single-cell transcriptomic analyses have revealed distinct malignant subpopulations co-expressing epithelial and mesenchymal markers that contribute to intratumoral heterogeneity and therapeutic resistance (15). Despite these insights, the optimal therapeutic strategy for MBC remains underexplored owing to its rarity and heterogeneity (16). In this study, we investigated the clinical characteristics and treatment approaches for MBC through a retrospective analysis of nine patients diagnosed at Hanzhong Central Hospital, aiming to summarize the diagnostic and therapeutic experiences in the context of emerging molecular insights.

## 2 Materials and methods

### 2.1 Data collection

Nine female patients diagnosed with MBC at Hanzhong Central Hospital between January 2016 and July 2025 were included in this retrospective analysis. Male patients were excluded. All the procedures involving human participants complied with the 2013 revision of the Declaration of Helsinki. Ethical approval was obtained from the Ethics Review Board of Hanzhong Central Hospital, Shaanxi Province, China (Approval No.: [202622]), which waived the need for individual informed consent for this retrospective study.

### 2.2 Inclusion and exclusion criteria

#### 2.2.1 Inclusion criteria

(1) Adult female patients with T1-4N0-3M0 disease (2); patients with a definitive pathological diagnosis confirmed postoperatively at our hospital; and (3) patients with complete medical records.

#### 2.2.2 Exclusion criteria

(1) bilateral breast cancer (2); recurrent breast cancer (3); metastatic breast cancer at the initial diagnosis (4); patients who received radiotherapy, chemotherapy, or endocrine therapy at other institutions prior to surgery; and (5) metaplastic carcinoma originating from the areola, nipple, or skin appendages.

### 2.3 Pathological assessment

The current diagnostic criteria for MBC rely primarily on conventional pathological and immunohistochemical techniques. To achieve a more precise tumor assessment, a comprehensive measurement of the total pathological size was performed, along with a detailed evaluation of the cytoplasmic extent, cystic changes, tumor necrosis, and calcification. A detailed documentation of the lymph node status of each patient was recorded.

According to the 2020 Chinese Society of Clinical Oncology (CSCO) guidelines, positivity thresholds were established at 1% for estrogen receptor (ER), progesterone receptor (PR), and androgen receptor (AR), and at 14% for Ki-67. The HER2 status was classified as positive based on either an immunohistochemical (IHC) score of 3+ (assessed using HercepTest; Dako, Carpinteria, CA, USA, according to the manufacturer’s instructions) or confirmed amplification via *in situ* hybridization (FISH) (17). All pathological slides were independently reviewed by two dedicated breast pathologists with more than 10 years of experience in breast pathology. Diagnoses were reassessed according to the 2019 World Health Organization (WHO) Classification of Tumours of the Breast (5). Cases with discordant interpretations were resolved by consensus discussion with a third senior pathologist. The histological subtypes were classified based on the predominant cellular component and the presence of heterologous mesenchymal elements, according to the WHO criteria for metaplastic breast carcinoma.

### 2.4 Statistical analysis

Given the descriptive nature of this retrospective case series and the limited sample size (n=9), no inferential statistical tests were performed. Descriptive statistics were used to summarize clinical and pathological characteristics. Continuous variables were reported as medians with ranges, and categorical variables were reported as frequencies with percentages. Survival outcomes were summarized using descriptive statistics. All analyses were conducted using SPSS version 26.0 (IBM Corp., Armonk, NY, USA). This retrospective case series was reported in accordance with the STROBE (Strengthening the Reporting of Observational Studies in Epidemiology) statement, where applicable.

## 3 Results

**Figure 1:**
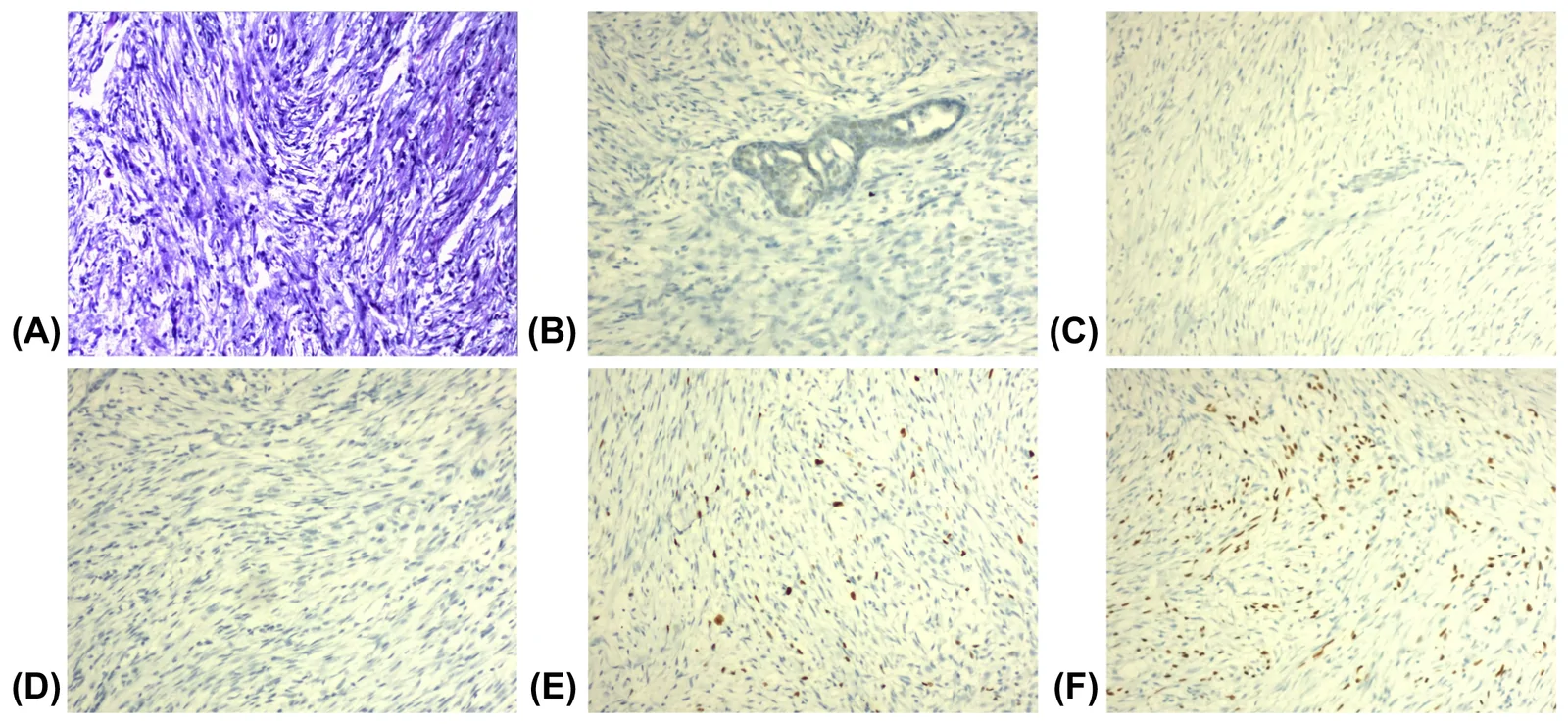
The pathological images of Patient 2. **(A)** Atypical spindle cells with marked nuclear atypia and diverse growth patterns. **(B)** ER; **(C)** PR; **(D)** HER2; **(E)** Ki67; and **(F)** P63. Bar: 100 μm. ER, estrogen receptor; PR, progesterone receptor; HER2, human epidermal growth factor receptor 2.

**Figure 2:**
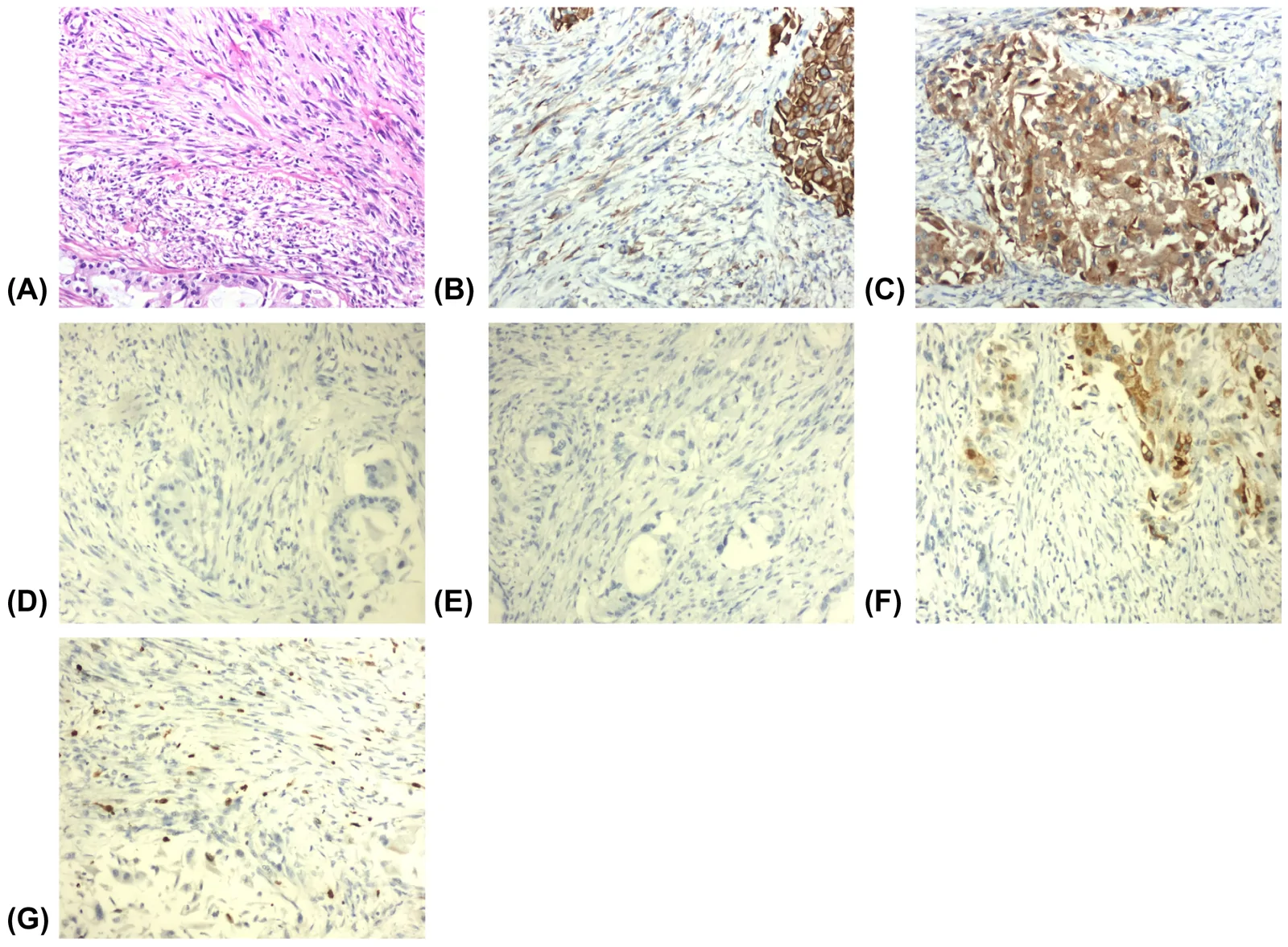
The pathological images of Patient 5. **(A)** Ductal structures are visible in the lower part of the image, while the upper part is composed entirely of spindle cells exhibiting marked cellular atypia. **(B)** Ckpan; **(C)** GCDFP15; **(D)** ER; **(E)** PR; **(F)** HER2; **(G)** Ki67. ER, estrogen receptor; PR, progesterone receptor; HER2, human epidermal growth factor receptor 2. Bar: 100 μm.

**Figure 3:**
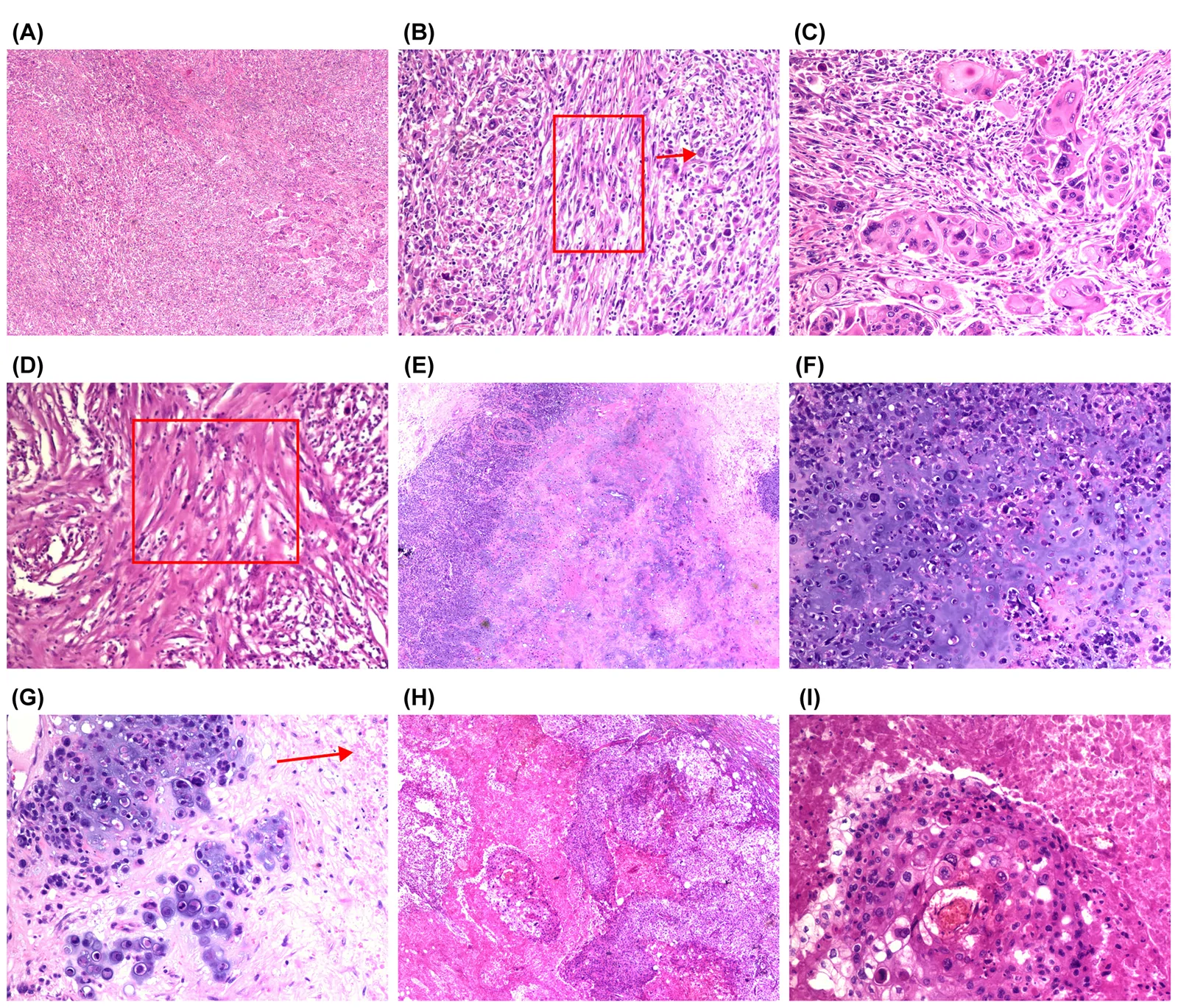
**(A)** The left panel demonstrates high-grade spindle cell carcinoma, and the right panel demonstrates squamous cell carcinoma (×20; H&E). **(B)** High-grade spindle cells arranged in long, interlacing fascicles (red box); tumor giant cells are readily identified (×100; H&E). **(C)** Squamous cells intermingled with spindle cells, with conspicuous atypical mitotic figures (arrows) (×100; H&E). **(D)** In other areas of the same patient, spindle cells exhibit only mild atypia and merge with a keloid-like stroma (red box) (×100; H&E). **(E)** Nodular growth pattern with a densely cellular periphery and a hypocellular, matrix-rich center (×20; H&E). **(F)** Myxochondroid metaplasia within the stroma adjacent to round carcinoma cells (×100; H&E). **(G)** Necrosis (arrow) is present to the right of the chondroid metaplasia (×100; H&E). **(H)** Sheets of non-keratinizing squamous cells with extensive tumor necrosis (box) (×20; H&E). **(I)** Medium-power view demonstrating single-cell keratinization (×100; H&E).

Demographic data, clinical and pathological characteristics, treatment modalities, and follow-up outcomes were retrospectively analyzed according to a predesigned protocol. Patient clinical and tumor characteristics are summarized in **Table 1**, while treatment details and outcomes are presented in **Table 2**. Histopathologically, the nine MBC cases exhibited diverse morphological features. Spindle cell carcinoma, characterized by fascicles of atypical spindle cells with variable mitotic activity, was the most common subtype (five of nine, 55.6%). Mixed-type metaplastic carcinoma displayed both spindle and squamous components; two of nine cases (22.2%) involved this subtype, with one case showing chondroid heterologous elements and another showing osseous differentiation. Squamous cell carcinoma demonstrated sheets of malignant squamous cells with intercellular bridges and keratinization and was observed in one of nine cases (11.1%). Low-grade adenosquamous carcinoma showed well-formed glandular structures admixed with solid squamous nests in a background of bland spindle cells and was also observed in one of nine cases (11.1%). Tumor necrosis was identified in six of nine cases (66.7%), and lymphovascular invasion was observed in four of nine cases (44.4%). The detailed histopathological features of each case are summarized in **Table 3**. Pathological images showing ER-positive expression (5%) in patient 2 and HER2 2+ in patient 5 are presented in **Figures 1** and **2**, respectively. Routine hematoxylin–eosin (H&E) staining and light microscopic evaluation were performed on all included tissue specimens. The tumor exhibited prominent histologic heterogeneity, with spindle cell carcinoma and squamous cell carcinoma components intermingled within the same lesion. Significant differences in the degree of cellular atypia, stromal remodeling and extent of tumoral necrosis were observed among different tumor regions. The typical microscopic pathological manifestations are shown in **Figure 3**.

**Table 1:** Clinicopathological characteristics of nine patients with metaplastic breast cancer.

No.	Age	Menopausal status	Tumor size (cm)	N stage*	Ki67 (%)	Histological type	ER	PR	HER2	AR
1	54	Post	3.5	0	30	Spcc	0	0	0	0
2	47	Pre	7.0	0	20	Spcc	5-10%	0	0	0
3	26	Pre	3.2	2	30	Spcc	0	0	2+/FISH(+)	0
4	44	Pre	3.8	2	70	Mtmc	0	0	0	0
5	49	Pre	1.5	0	30	Mtmc	0	0	2+/FISH (–)	+
6	56	Post	1.9	0	30	Spcc	0	0	1+	0
7	74	Post	1.6	0	60	Sqcc	0	0	0	0
8	53	Post	5.9	2	60	Lgac	0	0	0	0
9	76	Post	4.5	0	45	Spcc	0	0	0	0

ER, PR, AR, and HER2 positivity was determined based on at least one positive biopsy or postoperative pathology result. HER2 (2+) cases were confirmed using fluorescence *in situ* hybridization. *Tumor staging was based on the AJCC 8th edition. ^#^Patient 3 showed dynamic HER2 conversion: negative on initial core needle biopsy, converted to HER2-positive (2+/ISH+) following neoadjuvant chemotherapy, and reverted to negative upon disease recurrence. Pre, premenopausal; Post, postmenopausal; N stage based on preoperative pathological staging; ER, estrogen receptor; PR, progesterone receptor; AR, androgen receptor; HER2, human epidermal growth factor receptor 2; Spcc, spindle cell carcinoma; Sqcc, squamous cell carcinoma; Mtmc, mixed-type metaplastic carcinoma; Lgac, low-grade adenosquamous carcinoma.

**Table 2:** Treatment modalities and clinical outcomes.

No.	Surgery	Chemotherapy	Radiotherapy	DFS (months)	OS (months)	Metastasis site	Alive
1	MRM	AC-T	No	12	28	Liver	No
2	MRM	AC×4	No	14	32	Lung	No
3	C+ALND	TAC-HP	Yes	12	38	Liver	No
4	MRM	AC-T	Yes	10	23	Liver+Brain	No
5	M+SLNB	TC×6	No	30	30+	No	Yes
6	M+SLNB	AC-T	No	27+	27+	No	Yes
7	M+SLNB	AC-T	No	26	26+	No	Yes
8	MRM	TAC	Yes	19	31	Liver	No
9	M+SLNB	None	No	9	15+	Brain+Bone	Yes

Chemotherapy regimens: A, anthracycline; C, cyclophosphamide; T, taxane; H, trastuzumab; P, pertuzumab. “+” indicates that the patient is still alive at the time of the last follow-up and the follow-up is ongoing. Abbreviations: MRM, modified radical mastectomy; C+ALND, conservation surgery + axillary lymph node dissection; M+SLNB, mastectomy + sentinel lymph node biopsy; DFS, disease-free survival; OS, overall survival. The “+” sign following DFS and OS indicates censored cases during follow-up.

**Table 3:** Detailed histopathological features of nine patients with metaplastic breast cancer.

No.	Histological subtype	Spindle cell component (%)	Squamous differentiation	Heterologous elements	Tumor necrosis	Lymphovascular invasion	DCIS component	Margin status
1	Spindle cell carcinoma	>90%	Absent	None	Present	Absent	Absent	Negative
2	Spindle cell carcinoma	>90%	Focal	None	Present	Present	Present	Negative
3	Spindle cell carcinoma	95%	Focal	None	Present	Present	Absent	Negative
4	Mixed-type metaplastic carcinoma	40%	30%	Chondroid	Present	Present	Absent	Positive
5	Mixed-type metaplastic carcinoma	50%	20%	Osseous	Absent	Absent	Present	Negative
6	Spindle cell carcinoma	>90%	Absent	None	Present	Absent	Absent	Negative
7	Squamous cell carcinoma	5%	95%	None	Present	Absent	Absent	Negative
8	Low-grade adenosquamous carcinoma	None	30%	None	Absent	Absent	Present	Negative
9	Spindle cell carcinoma	95%	Focal	None	Present	Present	Absent	Negative

DCIS, ductal carcinoma *in situ*.

Of the nine cases, seven (77.8%) were triple-negative breast cancer, one (11.1%) was ER-low positive, and one (11.1%) was HER2-positive. Notably, in the HER2-positive case, HER2 status was negative on initial core needle biopsy, converted to positive following neoadjuvant chemotherapy, and subsequently reverted to negative upon disease recurrence. Dual HER2 blockade with trastuzumab and pertuzumab, which has demonstrated a survival benefit in HER2-positive breast cancer, was administered to this patient as targeted therapy (18).

Six patients received adjuvant chemotherapy and two received neoadjuvant therapy. Chemotherapeutic agents include cyclophosphamide, doxorubicin, and taxanes. One patient did not undergo adjuvant chemotherapy because of significant comorbidities.

Three patients received postoperative radiotherapy to the chest wall delivered via cobalt-60 gamma rays with bilateral tangential fields. A total dose of 50 Gy was prescribed in 25 fractions over five weeks. One patient (11%) developed brain metastasis postoperatively within nine months.

The median follow-up duration was 24 months (range: 15.7–28.4 months). At analysis, three patients were alive and disease-free, one was alive with distant metastasis, and five had succumbed to distant metastasis. The predominant sites of distant metastasis were the liver and brain.

Whole-brain radiotherapy was initiated in most patients with brain metastases; however, all but one patient subsequently died. Patient 9, who had both brain and bone metastases, received whole-brain radiotherapy following progression from bone involvement and remained alive at the time of last follow-up (OS 15+ months).

## 4 Discussion

### 4.1 Clinical and pathological characteristics

Compared to invasive breast cancer, MBC typically manifests at an older age and is more prevalent in postmenopausal women aged > 50 years (19). In this study, five of the nine participants were postmenopausal women over 50 years of age. MBC tumors are generally larger, painless, expansive, firm, and poorly mobile compared with typical breast cancers. Most patients seek medical attention because of palpable breast lumps. In the present series, only two of nine patients (22.22%) presented with tumors exceeding 5 cm in diameter, with a median tumor size of 3.66 cm (range: 1.5–7.0 cm). This trend may be attributed to enhanced awareness of breast self-examination among women and the widespread use of routine screening.

The accuracy of breast ultrasound evaluation depends on the physician’s technical expertise and equipment quality. Early studies indicated that MBC may manifest characteristics similar to those of benign breast tumors (20), whereas recent investigations have shown that MBC often presents features akin to malignant tumors, including irregular morphology, indistinct margins, and predominantly hypoechoic internal echoes (21). In this study, the ultrasonographic findings in all nine patients were consistent with malignant tumor characteristics. On mammography, MBC generally appears as high-density shadows with indistinct margins, typically without calcification.

One patient (Patient 5, 11.1%) in our cohort was AR-positive. Although AR is an active area of investigation in triple-negative breast cancer, including metaplastic subtypes, AR-targeted approaches were not explored in the present cohort owing to its retrospective nature and limited sample size. Future studies with larger cohorts are needed to clarify the prognostic and therapeutic implications of AR expression in MBC.

### 4.2 Literature context on molecular targets

Recent large-cohort genomic studies have identified frequent alterations in TP53 (73.2%) and PIK3CA (46.0%) in MBC, with PIK3CA mutations potentially associated with improved disease control in taxane-treated patients (15). Single-cell transcriptomic analyses have further revealed intratumoral heterogeneity with co-expression of epithelial and mesenchymal markers (15). In addition, preclinical data have suggested that combining PI3K inhibitors with NOS inhibitors may overcome chemotherapy resistance (22), leading to an ongoing phase II trial (NCT05660083) (23). Immunotherapy has also been explored, with the I-SPY2.2 trial suggesting potential benefit in immune-enriched subtypes (24). However, none of these molecular or targeted approaches were investigated in our cohort, and we did not perform genomic profiling on our patients. Therefore, the above discussion is provided strictly as background from the literature, and the described molecular data were not generated from our cohort.

### 4.3 Treatment sequencing considerations from the literature

The optimal sequence of surgery and systemic therapy for MBC remains controversial. The efficacy of neoadjuvant chemotherapy is limited, with generally low pathological complete response rates (approximately 4.9%–9.8%) (25, 26). A large-scale retrospective study using the National Cancer Database (2025) reported that, in operable MBC, upfront surgery combined with adjuvant chemotherapy was associated with improved overall survival (adjusted HR 0.69, 95% CI 0.59–0.8), whereas neoadjuvant chemotherapy showed no survival benefit at 1 or 3 years (27, 28). These data, derived from external cohorts, suggest that upfront surgery may be a reasonable approach for operable disease. In our series, the axillary lymph node metastasis rate was 33.3% (three of nine). Sentinel lymph node biopsy (SLNB) performed concurrently with tumor resection is therefore recommended, with the decision to perform further axillary dissection guided by SLNB findings. However, given the extremely limited sample size of our own cohort, we cannot draw any therapeutic conclusions from our data, and all treatment considerations discussed here are derived from larger retrospective studies.

### 4.4 Study limitations

Some limitations of this study should be acknowledged. First, the sample size (n=9) is extremely small, reflecting both the rarity of MBC and the single-center, retrospective design. Although this cohort represents the complete dataset of MBC cases at our institution over a ten-year period, our findings should be considered hypothesis-generating rather than conclusive. Second, the study lacks comprehensive molecular or genomic profiling (e.g., PIK3CA/TP53 mutation testing, PD-L1 expression, or next-generation sequencing), precluding any correlation between molecular alterations and clinical outcomes. Third, breast MRI data were unavailable for most patients, limiting radiological correlation. Fourth, the median follow-up of 24 months is relatively short for assessing long-term survival in an aggressive malignancy; longer follow-up is needed to capture late recurrences. Fifth, none of our patients received the emerging targeted or immunotherapeutic agents currently under investigation (29), limiting direct comparisons with contemporary treatment approaches. We have initiated prospective enrollment and continued follow-up at our center and encourage future multicenter collaborations to assemble larger cohorts with integrated genomic characterization.

## 5 Conclusions

This retrospective series of nine MBC cases highlights the diagnostic pitfalls, profound pathological heterogeneity, and aggressive natural history of this rare breast cancer subtype. Preoperative diagnosis remains challenging and continues to depend on postoperative histopathological and immunohistochemical evaluation. The high rate of liver and brain metastases observed in our cohort underscores the urgent need for more effective systemic therapies. However, the optimal treatment sequence—particularly the role of neoadjuvant versus adjuvant chemotherapy—remains undefined and requires validation in larger, prospective cohorts.

Given the significant limitations of our single-center data, including the small sample size, short follow-up, and lack of molecular profiling, we emphasize that robust conclusions cannot be drawn from this study alone. Our findings are intended to serve as a hypothesis-generating foundation for future investigations. We strongly advocate for prospective, multicenter registries incorporating comprehensive clinical annotation and genomic characterization to guide the development of evidence-based treatment strategies for this challenging disease.

## Data Availability

The raw data supporting the conclusions of this article will be made available by the authors, without undue reservation. Due to patient privacy and institutional policy, the full dataset is not publicly archived but is available from the corresponding author upon reasonable request.
